# The association between early childhood onset epilepsy and attention-deficit hyperactivity disorder (ADHD) in 3237 children and adolescents with Autism Spectrum Disorder (ASD): a historical longitudinal cohort data linkage study

**DOI:** 10.1007/s00787-022-02041-3

**Published:** 2022-08-04

**Authors:** Lauren Carson, Valeria Parlatini, Tara Safa, Benjamin Baig, Hitesh Shetty, Jacqueline Phillips-Owen, Vibhore Prasad, Johnny Downs

**Affiliations:** 1 Department of Psychological Medicine, Institute of Psychiatry, Psychology and Neuroscience, King’s College London, London, UK; 2 Department of Child and Adolescent Psychiatry, Institute of Psychiatry, Psychology and Neuroscience, King’s College London, London, UK; 3 National Institute for Health Research (NIHR) Biomedical Research Centre, South London and Maudsley NHS Foundation Trust, London, UK; 4 School of Population Health and Environmental Sciences, King’s College London, London, UK

**Keywords:** Childhood epilepsy, ASD, ADHD, Data Linkage

## Abstract

Children and young people with Autism Spectrum Disorder (ASD) have an increased risk of comorbidities, such as epilepsy and Attention-Deficit/Hyperactivity Disorder (ADHD). However, little is known about the relationship between early childhood epilepsy (below age 7) and later ADHD diagnosis (at age 7 or above) in ASD. In this historical cohort study, we examined this relationship using an innovative data source, which included linked data from routinely collected acute hospital paediatric records and childhood community and inpatient psychiatric records. In a large sample of children and young people with ASD (*N* = 3237), we conducted a longitudinal analysis to examine early childhood epilepsy as a risk factor for ADHD diagnosis while adjusting for potential confounders, including socio-demographic characteristics, intellectual disability, family history of epilepsy and associated physical conditions. We found that ASD children and young people diagnosed with early childhood epilepsy had nearly a twofold increase in risk of developing ADHD later in life, an association which persisted after adjusting for potential confounders (adjusted OR = 1.72, CI95% = 1.13–2.62). This study suggests that sensitive monitoring of ADHD symptoms in children with ASD who have a history of childhood epilepsy may be important to promote early detection and treatment. It also highlights how linked electronic health records can be used to examine potential risk factors over time for multimorbidity in neurodevelopmental conditions.

**Supplementary Information:**

The online version contains supplementary material available at 10.1007/s00787-022-02041-3.

## Introduction

Autism Spectrum Disorder (ASD) is a complex neurodevelopmental disorder, which is thought to affect approximately 1–2% of the general population, although the prevalence may vary according to the country, age and assessment criteria [1–3]. ASD is a highly heterogenous disorder with regard to its clinical presentation, aetiology, underlying neurobiology, and degree of severity [4]. Further, as reported in Simonoff, Pickles [5], 70% of children with ASD have at least one comorbid disorder, with 41% having two or more disorders [5]. Two of the most common comorbid conditions are epilepsy, with rates varying from 5 to 39% [6]; and Attention-Deficit/Hyperactivity Disorder (ADHD), with co-occurrence rates varying from 28 to 31% [5, 7].

Despite evidence showing that ASD is associated with both higher rates of epilepsy and ADHD, their potentially common underlying biological mechanisms and temporal relationship remain unclear due to the lack of longitudinal studies, which means that important clinical questions remain unanswered. Prior studies suggest that epilepsy is associated with increased rates of ADHD or ASD, relative to the general population, suggesting these conditions may be linked to common underlying pathogenic pathways [8, 9]. Further, as Robinson [10] suggested, there may be a relationship between childhood onset epilepsy in children with ASD and the timing of later psychiatric/behavioural problems. However, as Boothe and Zuna [11] highlighted in their review, there is a lack of evidence on the developmental course of ASD, epilepsy and ADHD: cross-sectional designs, with small convenience samples, and inconsistent definitions of the neurological and neurodevelopmental disorders cannot provide adequate evidence and conclusive findings. Therefore, it is not clear whether there is a temporal association between epilepsy and ADHD for children with ASD. Further, other studies have suggested that neurodevelopmental conditions may be associated with greater rates of epilepsy. For example, children with ASD and intellectual disability (ID) have greater rates of epilepsy compared to ASD children without ID [12, 13], and lower cognitive ability is an independent risk factor for epilepsy in ASD (Viscidi et al., 2013). Also, a study by Ewen, Marvin [14] showed that several neurodevelopmental vulnerabilities, including the presence of ID, language atypicalities, ASD‐specific symptoms severity, and motor skill abnormalities, all independently predicted an increased risk for epilepsy. To better understand the relationship between epilepsy and risk of ADHD among children with ASD, we aimed to ascertain whether there was a temporal sequence between epilepsy incidence and risk of subsequent ADHD diagnosis. Using a longitudinal design, this study investigated the association between epilepsy diagnosed before the age of 7 and subsequent risk of ADHD among children with ASD, using electronic health records (EHRs).

EHRs provide an opportunity to perform longitudinal analyses to explore the association of different disorders, including relatively rare ones, which are otherwise difficult to study because even large population surveys would only detect small numbers. EHRs are effective to overcome this limitation, especially for disorders such as epilepsy, ASD and ADHD that are so severe that clinical contact is almost inevitable [15].

Based on the earlier work, we hypothesised that in a cohort of children with ASD, early onset epilepsy would be associated with a greater risk for later ADHD. To test this and address the limitations of prior studies, we conducted a historical cohort study in a large sample of children with ASD and examined the association between early childhood onset epilepsy (before age 7) and later ADHD diagnosis (at age 7 or above). We used an innovative data source, which included linked data from hospital paediatric and psychiatric records.

## Methods

### Data sources

We used data obtained from the EHR database of the South London and Maudsley NHS Foundation Trust (SLaM) linked to Hospital Episode Statistics (HES) [16–19]. SLaM is one of the biggest suppliers of mental healthcare in Europe, providing specialist services to 1.2 million residents in London [20]. In 2007, SLaM developed the Clinical Record Interactive Search (CRIS) tool to form an extensive, anonymised, de-identified database permitting secondary research into mental health [16]. To date, it has been used in over 150 published studies including Child and Adolescent Mental Health Services (CAMHS) focussed research within ASD and ADHD clinical populations [18, 20–24]. In this study, the CRIS system was used to explore structured and free-text fields of the clinical records of the over 35,000 children and young people accessing SLaM services [16]. Structured fields provide demographic and clinical information, e.g. the diagnosis of neurodevelopmental disorders according to the International Classification of Diseases 10th edition (ICD‐10) criteria [25]. Natural language processing approaches, based on Generalised Architecture for Text Engineering (GATE), were used to extract information from free text, such as clinical notes [20].

We extracted information on epilepsy episodes recorded between 1997 and 2013 from the HES, which is the central repository for all routinely collected secondary care data held by NHS Digital. This provides clinical, background and geographical information for all NHS admissions, emergency department and outpatient visits, even for private patients in NHS hospitals in England and Wales. Hospital admission episodes were recorded within structured fields in the HES records, and each of them was associated with a discharge diagnosis according to ICD‐10 criteria [25]. A linkage between CRIS and HES datasets was generated by NHS Digital.

### Study design and sample

We used a retrospective longitudinal open-cohort design including children and young people (aged 3–18 years) receiving care in SLaM between 1st January 2008 and 31st March 2013 and diagnosed with ASD (including autism, ASD, Asperger’s Syndrome, and Pervasive Developmental Disorder) in accordance with ICD-10 classifications (F84.0, F84.1, F84.5, F84.9). This was a dynamic clinical cohort, as participants entered and withdrew at varying time points according to their clinical care. Within this ASD cohort, we identified those with a diagnosis of early onset epilepsy before age 7 using HES linked data (i.e. any epilepsy diagnosis before age 7 recorded between 1997 and 2013). Later diagnoses for ADHD (i.e. occurring at age 7 or above) were extracted from CRIS records between 1st January 2008 and 31st March 2013. We designed the study to ensure separation in time between exposure and outcome detection. This reduced the potential of ‘reverse causality’ as an alternative explanation for any associations we may have found between epilepsy and ADHD. We used an early childhood age range cut-off for epilepsy (i.e. diagnosis between 0 and 6 years) to capture the peak incident age periods of epilepsy of childhood, and hence maximise the opportunity for identification of our main exposure of interest [26]. We applied a 7 and above age range for ADHD outcome detection as average age of ADHD diagnosis in Europe ranges between 6.2 and18.1 years [27].

### Main exposure and outcome

Hospital admission with early childhood epilepsy was defined as one or more HES records containing ICD-10 diagnosis codes G40 ‘Epilepsy’ and/or G41 ‘Status epilepticus’ occurring before the child’s 7th birthday. Epilepsy was diagnosed by specialists working in secondary care according to the NICE guidelines (https://www.nice.org.uk/guidance/cg137). Prior studies indicated that epilepsy can be reliably identified from medical records with specificity and sensitivity above 90% [28].

ADHD diagnoses were identified from both structured and unstructured diagnosis fields within CRIS, using classification terms according to ICD-10 criteria Hyperkinetic, other Hyperkinetic Disorders, Attention-Deficit Disorder, Hyperkinetic Conduct Disorder (F90.0, 90.1, 90.8, 90.9). Unstructured ADHD diagnoses were extracted using previously established natural language processing techniques to detect diagnosis within CRIS [17], which have a positive predictive value of 0.86 for neurodevelopmental disorders [29]. ADHD diagnoses were made by child psychiatrists, paediatricians or specialist psychologists with training and expertise in ADHD, based on standardised questionnaires, clinical interviews and direct surveillance in home, school and clinical contexts. Data on ADHD diagnosis was available on CRIS for all included children with ASD until they remained active patients or the end of the study.

### Covariates

This study aimed to identify potential independent associations between idiopathic early onset epilepsy and later ADHD diagnosis. We therefore controlled for potential risk factors for non-idiopathic epilepsy and ADHD. We considered known genetic and structural/metabolic aetiological risk factors recognised by the International League Against Epilepsy (ILAE) Commission on Classification and Terminology [30]. These consisted of central nervous system (CNS) infections; neoplasms; autoimmune conditions; endocrine, nutritional, metabolic, and neurodegenerative diseases; perinatal complications; congenital malformations; chromosomal abnormalities; and head injuries. In addition, prior physical health complications were also identified [31]. These data were extracted using their respective ICD10 codes from the HES records as recorded up to age 7 (Table S1). Further, CRIS data provided information on additional potential confounders including sex, age at first coding of ASD diagnosis in the CAMHS records, comorbid ID (ICD-10 F7x), family history of epilepsy, ethnicity, and neighbourhood levels of socio-economic deprivation (see Supplementary material).

### Statistical analysis

**Fig. 1:**
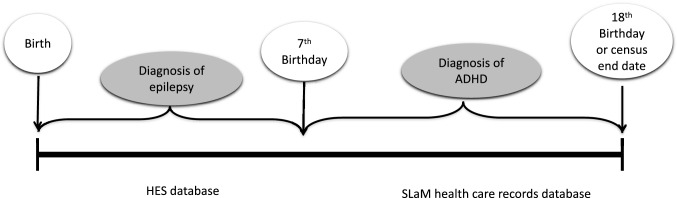
Study design and timing of exposure (epilepsy diagnosis) and outcome (ADHD diagnosis) data extraction

Figure 1 provides a visual representation of the longitudinal study design. To estimate the risk of early childhood epilepsy (before age 7) for a later ADHD diagnosis (age 7 or above), we excluded children who had an ADHD diagnosis recorded prior to age 7. Epilepsy and ADHD diagnosis were coded as binary variables (presence vs absence). The statistical analyses were conducted using Stata version 14 (StatCorp, College Station, Texas, USA).

We first conducted descriptive statistics to identify demographic and clinical characteristics of our ASD population according to the presence/absence of a diagnosis of either early childhood epilepsy or ADHD. Then, we investigated the relationship between childhood epilepsy (before age 7) and ADHD diagnosis (age 7 or above) by conducting a set of logistic regressions, which adjusted for potential confounders, including socio-demographic characteristics, intellectual disability, family history of epilepsy and associated physical conditions [31]. Rare physical disorder exposures e.g. tumours, were combined in the multivariable analyses, and collapsed into a single binary variable (presence vs absence of any physical confounder).

## Results

Table 1 shows the characteristics of the study population with and without hospitalisation for early childhood epilepsy. Of the 3237 children who were diagnosed with ASD, 121 (3.7%) were diagnosed with epilepsy before the age of 7 years. There was a higher proportion of females in the epilepsy vs no-epilepsy group (36.0 vs 21.6%), who had a twofold greater risk of epilepsy compared to males (OR = 2.23, CI95% = 1.53–3.25). The median duration of follow-up with child mental health services was 14.2 months [Interquartile range 2.04–46.8 months]. Children with ASD in the epilepsy group encountered child mental health services at a younger age (mean age 10.1 vs 11.1 years). ADHD diagnosis was more prevalent in the epilepsy group (37.2% vs 24.6%). As expected, rates of epilepsy were much higher in ASD children with additional physical health or intellectual disorders.

**Table 1:** Sample characteristics by history of early childhood epilepsy (< 7 years)

ASD SAMPLE CHARACTERISTICS	No epilepsy *N*(%) 3116 (96.3)	With epilepsy *N*(%) 121 (3.7)
ADHD diagnosis, *N* (%)	765 (24.6)	45 (37.2)
Female, *N* (%)	673 (21.6)	46 (36.0)
Age at ASD diagnostic coding, mean (SD)	11.13 (3.78)	10.1 (4.1)
Age group *N* (%)		
3–6 years	309 (9.92)	23 (19.0)
6–12 years	1460 (46.85)	55 (45.5)
12–18 years	1347 (43.91)	43 (35.5)
Ethnicity, *N* (%)		
White British	1468 (47.11)	73(60.3)
White other	142 (4.56)	≤ 5^a^
East Asian/British East Asian	57 (1.83)	≤ 5^a^
Black African/British Black African	593 (19.03)	16 (13.2)
African-Caribbean/British African-Caribbean	116 (3.72)	≤ 5^a^
Mixed	343 (11.01)	12 (9.9)
South Asian/ British South Asian	76 (2.44)	≤ 5^a^
Other	321 (10.30)	11 (9.1)
Neighbourhood deprivation, *N* (%)		
1st (least deprived)	718 (23.40)	35 (28.9)
2nd	764 (24.89)	29 (24.0)
3rd	796 (25.94)	28 (23.1)
4th (most deprived)	791 (25.77)	29 (24.0)
Intellectual disability, *N* (%)	576 (18.5)	71 (58.7)
Family history of epilepsy, N (%)	96 (3.0)	≤ 5^a^
Physical risk factors, *N* (%)		
CNS Infection	10 (.3)	≤ 5^a^
Any Tumour	≤ 5^a^	≤ 5^a^
Autoimmune Disease	≤ 5^a^	≤ 5^a^
Endocrine/metabolomic disorder	26 (0.8)	≤ 5^a^
CNS Myodegeneration	53 (1.7)	33 (38.4)
Perinatal Complications	220 (7.1)	18 (14.9)
Congenital Malformations	123 (4.0)	38 (31.4)
Head Injury	155 (5.0)	18 (14.9)
CNS Infection	≤ 5^a^	≤ 5^a^
Any physical of the above physical factors	412 (13.2)	73 (60.3)

^a^actual numbers suppressed to reduce risk of statistical disclosure

Table 2 shows the characteristics of the ASD sample by ADHD outcomes. Of the 3237 children in this cohort, we found 810 had an additional diagnosis of ADHD (25.0%). The ADHD group compared to the non-ADHD group, had much lower prevalence of females (24.1% vs 16.4%), were more likely to be seen by CAMHS between 6 and 12 years, and were more often of white ethnicity. The two groups had similar levels of neighbourhood deprivation. The prevalence of early childhood epilepsy was higher in the ADHD group (5.6% vs 3.1%). Furthermore, several physical health conditions including hospitalisation with head-injury occurring before 7, perinatal complications and endocrine-metabolic disorders were associated with increased risk of ADHD (also see Table 3).

**Table 2:** Sample characteristics by ADHD outcome (> 7 years)

ASD sample characteristics	No ADHD *N*(%) 2427 (75.0)	With ADHD *N*(%) 810 (25.0)
Early childhood onset epilepsy, *N* (%)	76 (3.1%)	45(5.6%)
Female, *N* (%)	586 (24.1)	133 (16.4)
Age at ASD diagnostic coding, mean (SD)	11.22 (3.7)	10.7 (3.60)
Age group *N* (%)		
3–6 years	261 (10.8)	71 (8.77)
6–12 years	1085 (44.7)	430 (53.1)
12–18 years	1081 (44.5)	309 (22.2)
Ethnicity, *N* (%)		
White British	1083 (44.6)	458 (56.5)
White other	108 (4.5)	36 (4.4)
East Asian/ British East Asian	48 (2.0)	9 (1.1)
Black African/ British Black African	495 (20.4)	114 (14.1)
African-Caribbean/ British African-Caribbean	88 (3.6)	31 (3.8)
Mixed	259 (10.7)	96 (11.9)
South Asian/ British South Asian	66 (2.7)	14 (1.7)
Other	280 (11.5)	52 (6.42)
Neighbourhood deprivation, *N* (%)		
1st (least deprived)	536 (22.4)	217 (27.2)
2nd	572 (23.9)	221 (27.6)
3rd	645 (27.0)	179 (22.4)
4th (most deprived)	638 (26.7)	182 (22.8)
Intellectual disability, *N* (%)	471 (19.4)	176 (21.7)
Family history of epilepsy, *N* (%)	77 (3.2)	24 (3.0)
Physical risk factors, *N* (%)		
CNS Infection	13 (.54)	≤ 5^a^
Any Tumour	≤ 5^a^	≤ 5^a^
Autoimmune Disease	≤ 5^a^	≤ 5^a^
Endocrine/metabolomic disorder	≤ 5^a^	14 (1.7)
CNS Myodegeneration	36 (1.5)	13 (1.6)
Perinatal Complications	153 (6.3)	85 (10.5)
Congenital Malformations	113 (4.7)	48 (5.9)
Head Injury	60 (2.5)	35 (4.3)
CNS Infection	77 (3.2)	24 (3.0)
Any physical of the above physical factors	325 (13.4)	160 (19.8)

^a^actual numbers suppressed to reduce risk of statistical disclosure

**Table 3:** The longitudinal association between early childhood epilepsy and ADHD outcomes in children with ASD with incremental adjustment for potential confounders

ADHD Outcome	adjusted for socio-demographic factors Odds Ratio (aOR) (95% CI)	+ familial epilepsy aOR (95% CI)	+ intellectual disability aOR (95% CI)	+ physical confounders aOR (95% CI)
Epilepsy (< 7 years)	2.08 (1.39–3.11)	2.08 (1.39—3.10)	1.96 (1.30–2.96)	1.72 (1.13–2.62)
Sex (female)	0.605 (0.486–0.752)	0.607 (0.488–0.755)	0.603 (0.484–0.750)	0.598 (0.480–0.744)
Age at ASD coding				
3–6 years	Reference	Reference	Reference	Reference
6–12 years	3.54 (2.33–5.38)	3.54 (2.33–5.38)	3.53 (2.32–5.37)	3.59(2.36–5.46)
12–18 years	2.46 (1.61–3.76)	2.46 (1.61–3.77)	2.44 (1.59–3.74)	2.55 (1.66–3.92)
Ethnicity				
White British	Reference	Reference	Reference	Reference
White other	0.865 (0.576–1.30)	0.860 (0.572–1.29)	0.864 (0.573–1.29)	0.871 (0.579–1.31)
East Asian/ British East Asian	0.383 (0.171–0.860)	0.379 (0.169–1.29)	0.376 (0.167–0.845)	0.385 (0.171–0.865)
Black African/ British Black African	0.584 (0.452–0.755)	0.582 (0.450–0.751)	0.576 (0.445–0.744)	0.588 (0.455–0.761)
African-Caribbean/ British African-Caribbean	0.789 (0.492–1.26)	0.783 (0.488–1.25)	0.780 (0.486–1.25)	0.789 (0.492–1.27)
Mixed	0.891 (0.676–1.17)	0.885 (0.672–1.17)	0.882 (0.669–1.16)	0.890 (0.675–1.17)
South Asian/ British South Asian	0.536 (0.295–0.973)	0.532 (0.293–0.966)	0.525 (0.289–0.954)	0.529 (0.292–0.961)
Other	0.457 (0.329–0.636)	0.455 (0.327–0.632)	0.451 (0.324–0.628)	0.450 (0.323–0.626)
Neighbourhood deprivation				
1st (least deprived)	Reference	Reference	Reference	Reference
2nd	0.968 (0.766–1.22)	0.968 (0.766–1.22)	0.973 (0.769–1.23)	0.972 (0.769–1.23)
3rd	0.737 (0.577–0.942)	0.736 (0.576–0.941)	0.741 (0.580–0.948)	0.754 (0.589–.964)
4th (most deprived)	0.713 (0.555–0.918)	0.714 (0.555–.919)	0.720 (0.559–926)	0.725 (0.563–0.933)
Family history of epilepsy	–	0.768 (0.456–1.29)	0.764 (0.454–0.129)	0.754 (0.447–1.27)
Intellectual disability	–	–	1.15 (0.930–1.43)	1.10 (0.884–1.36)
Any physical confounders	–	–	–	1.40 (1.10–1.77)

Finally, we calculated odds ratios (OR) for the association between epilepsy and ADHD diagnosis (Table 3). The unadjusted OR was 1.82 (95% CI = 1.25–2.65). In the model adjusted for sex, age at diagnosis, ethnicity, deprivation scores, familial epilepsy, ID and other epilepsy-related confounders, the OR was 1.72 (CI95% = 1.13–2.62), which indicated that children with ASD that received a diagnosis of epilepsy before age 7 were almost twice as likely to be diagnosed with ADHD at or after age 7.

## Discussion

### Summary of findings

To the best of our knowledge, this is the first study to examine whether early childhood onset epilepsy is associated with a greater risk of mid childhood onset ADHD in children with ASD. We found a near twofold increase in risk of developing ADHD for ASD children if they had been hospitalised with epilepsy before the age of 7, an association which still persisted once associated physical conditions, family history, and ID were accounted for.

Previous studies have identified children with epilepsy as at increased risk for neurodevelopmental disorders. For instance, Reilly et al. (2019) reported that children with epilepsy before age 7 were at high risk for reaching clinical thresholds for ADHD (41%) or ASD (18%) [32]. Our study extends these findings using longitudinally collected data within a large ASD clinical sample. We showed that early childhood onset epilepsy had an association with a later clinical record of ADHD diagnosis. Furthermore, by adjusting for known potential causes of epilepsy and ADHD (such as neurodegenerative, metabolic and other conditions listed in Table S1), our findings suggest that idiopathic epilepsy may be an important risk factor for the ADHD phenotype within ASD. As observational, this study cannot definitely prove that early onset epilepsy causes mild childhood ADHD. However, we hope these novel findings encourage future studies to further test this hypothesis, e.g. by comparing the risk of ADHD in well-controlled longitudinal cohorts of ASD children with and without epilepsy. Various theories have been proposed to explain the association between idiopathic epilepsy and ADHD, which include a shared genetic basis causing underlying brain abnormalities, deregulation of the noradrenergic system, a common psychosocial environment, the effect of seizures and antiseizure medication use [33]. However, evidence focussing on the impact of other neurodevelopmental abnormalities in the occurrence of co-morbid epilepsy and ADHD is still limited [34]. Lo-Castro and Curatolo [8] suggest that there is evidence of a co-occurrence of an epilepsy/ASD phenotype or an epilepsy/ADHD phenotype that has a complex and heterogeneous pathogenesis, resulting from several altered neurobiological mechanisms involved in early brain development, and influencing synaptic plasticity, neurotransmitter transmission and functional connectivity. It is likely that rare clinically relevant genetic abnormalities, in addition to other social and environmental factors, may confer an increased risk for ASD/ADHD associated with epilepsy [35, 36].

Other findings of interest include the hospitalisation rates of epilepsy in children with ASD, which was 3.7% of the sample diagnosed before the age of 7 years. Past studies suggest a lifetime prevalence between 2.7% and 44.4% [37–39], thus our results are in the lower range relative to previous estimates. This is likely due to the narrow time window of early childhood to determine epilepsy diagnoses. In addition, we coded hospitalised cases of epilepsy only, thus excluded lower severity cases that may be exclusively managed in the community. Also, of interest is our finding that females were over twice as likely to have epilepsy as compared to males with ASD. This is in line with previous studies reporting that females with ASD tend to be more severely mentally disabled [12]. Nonetheless our findings are consistent with a recent meta-analysis by Lax-Pericall, Bird [40], which suggests that females may have a greater likelihood of shared ASD-epilepsy pathogenesis than males. Further, ID is also highly co-morbid with both ASD [41] and epilepsy [42], and ID is associated with an increased risk for both epilepsy and ASD.

Within our large clinical sample of 3237 children with ASD, 25% were diagnosed with ADHD after their 7th birthday. This is lower than findings from whole population Scandinavian register studies, which found within a sample of 28,468 individuals with ASD (age range 3–30), nearly 50% had ADHD [43]. However our findings were consistent with similar paediatric age community samples and study observation periods for detecting diagnosis, where 31% of the sample met full ADHD criteria [7]. This study also demonstrates that the medical risk factors associated with ADHD from the general population, including head-injury, perinatal complications, and endocrine/metabolic disorders, are consistent with the risk factors observed in the ASD population. Our multivariable model demonstrates that early onset epilepsy is a risk factor for later ADHD diagnosis even when these physical risk factors are controlled for.

When examining the demographics of the cohort, our model might suggest that belonging to non-White British ethnic groups or residing in more deprived areas may be associated with a lower risk of ADHD diagnosis. However, caution is warranted when interpreting these results, as ethnicity and socio-economic deprivation were included as confounders, and their estimates of effect should not be intended as casual. Further, these results could potentially reflect barriers in receiving health care. Further research is needed within diverse ethnic populations of ASD patients to explore how patient ethnicity and social background may affect diagnostic practices in CAMHS.

### Strengths and limitations

This study has several strengths. First, we analysed a clinical cohort of children with neurodevelopmental disorders including both community and inpatient CAMHS settings; therefore, this represents a ‘real world’ clinical population and results may generalise to other clinical populations. Second, our large sample size led to more precision when assessing the associations between exposures and outcomes. Third, we were able to examine the association between childhood epilepsy and ADHD diagnosis in longitudinally collected data. Finally, we measured outcomes associated with epilepsy up to five years after the initial diagnosis of ASD, a longer observation period than that of other comparable studies.

Limitations should also be considered. First, the diagnosis of epilepsy and that of the associated physical risk factors were reliant on clinical observations within a hospital setting, rather than a gold standard diagnostic assessment. Second, we identified epilepsy cases from hospital records only, therefore we may have missed those exclusively investigated and managed in the community, e.g. with lower severity epilepsy. Similarly, we only included children with ASD that were open cases in our CAMHS, thus we may have potentially excluded more severely ill children that were diagnosed with epilepsy but died before a diagnosis of ADHD could be made. Hence the sample may not fully represent the full range of children with ASD who received a diagnosis of early onset epilepsy. However, under-recording children with epilepsy may increase the rate of false negatives, which could underestimate (not overestimate) the strength of the association between epilepsy and ADHD comorbidity. Third, within the HES data there is limited information on the severity and type of epilepsy syndrome, hence we could only provide an average effect of epilepsy on ADHD, but different epilepsy syndromes may have a differential association with ADHD. Fourth, despite this being one of largest cohort studies of ASD in childhood, it may have been underpowered to control for some of the rarer physical health conditions associated with epilepsy/ADHD. Fifth, as this is an observational study, residual (known and unknown) confounders could represent a potential explanation for the association between epilepsy and ADHD. For example, we were unable to include medication use or family history of ASD/ADHD among confounders and therefore to estimate their effects on our results. However, these effects are likely to be small. Prior findings show that an association between epilepsy and ADHD persists following adjustment for family history of psychiatric disorders and in medication naïve groups [44]. For instance, a population-based case–control study showed that ADHD was 2.5-fold more common among children with newly diagnosed seizures than among controls [45]. Furthermore, as anti-epileptic medications would be on the pathway from the exposure to the outcome, adjustment would pose the risk to underestimate the total effect of epilepsy on ADHD. Finally, it is possible that children with ASD and epilepsy may have been under increased clinical scrutiny, which may potentially lead to a greater awareness of their co-morbidities, hence an increased rate of ADHD diagnoses. Unfortunately, these potential biases in clinical classification cannot be fully accounted for.

### Implications and future directions

Our findings suggest that children who present to mental health services with a history of early childhood epilepsy and ASD may also have ADHD. Clinicians should therefore be conscious of attributing a child’s hyperactive or inattentive behaviours to their ASD only, especially in the context of early childhood epilepsy. Early consideration of ADHD co-morbidity in children with ASD and early onset epilepsy could promote better detection and interventions, which are desirable as ADHD treatment in epilepsy is associated with improved outcomes [46]. General practitioners/family physicians should also be aware of ADHD symptoms being more prominent during primary school, especially in children known to have epilepsy and ASD. Finally, more comprehensive assessments of medical history should be undertaken as early childhood epilepsy (especially if occurring and remitting in the first year of life) may not be volunteered by the patient or parent/carer without specific probes offered at initial consultation. Our study requires replication, ideally using both large scale retrospective cohorts based on electronic health records and prospective cohort studies within clinical and general populations. Longitudinal studies investigating brain development are needed to clarify the aetiological pathways to the combined ASD-ADHD phenotypes and their potential treatment targets. For example, it is still unclear what potential genetic components underpin ASD-ADHD epilepsy subtypes [47]. Furthermore, future studies are needed to investigate whether antiseizure medication may play a role in increasing ADHD risk. To minimise the effects of confounding by indication, these studies should be conducted in an ASD-epilepsy only cohort and include detailed information on epilepsy type and severity. In sum, further longitudinal studies are needed to enable us to understand how epilepsy may be associated with attentional, hyperactive and impulsivity related impairments within ASD, and what treatments may help these impairments.

## Conclusions

We provide evidence that in children with ASD, early childhood onset epilepsy increases the risk of ADHD. This association remained following adjustment for sex, age, socio-economic deprivation, ethnicity, ID, family history of epilepsy or associated physical conditions. These findings are relevant for clinicians, as they illustrate the importance of various neurodevelopmental factors in driving the association between ASD-ADHD and epilepsy and extends our knowledge in this complex field. Although our results are derived from a clinical setting, and may not be directly applicable to the general population, they suggest that clinicians and caregivers of children with ASD may need to consider screening for potential ADHD early in those who have a history of childhood epilepsy. Earlier detection and access to evidence-based ADHD treatment may substantially improve the prognosis for children with these vulnerabilities.

### Supplementary Information

Below is the link to the electronic supplementary material.

Supplementary file1 (PDF 342 kb)

## Data Availability

Data are available on reasonable request. The data accessed by CRIS remain within an NHS firewall and governance is provided by a patient-led oversight committee. Access to data is restricted to honorary or substantive employees of the South London and Maudsley NHS Foundation Trust and governed by a local oversight committee who review and approve applications to extract and analyse data for research. Subject to these conditions, data access is encouraged and those interested should contact RS (robert.stewart@kcl.ac.uk), CRIS academic lead.

## Code availability

N/A.
