# Supplementary material for: The association between early childhood onset epilepsy and attention-deficit hyperactivity disorder (ADHD) in 3237 children and adolescents with Autism Spectrum Disorder (ASD): a historical longitudinal cohort data linkage study
Source: Eur Child Adolesc Psychiatry. 2022 Aug 4;32(11):2129–38. doi: 10.1007/s00787-022-02041-3 (PMC10576710; doi:10.1007/s00787-022-02041-3)
Supplement: Supplementary file 1 — Supplementary file1 (PDF 342 kb) [file 787_2022_2041_MOESM1_ESM.pdf]

Supplementary material

THE ASSOCIATION BETWEEN EARLY CHILDHOOD ONSET EPILEPSY AND ATTENTION-DEFICIT HYPERACTIVITY DISORDER (ADHD) IN 3237 CHILDREN AND ADOLESCENTS WITH AUTISM SPECTRUM DISORDER (ASD): A HISTORICAL LONGITUDINAL COHORT DATA LINKAGE STUDY. ECAP.

Lauren Carson[1], Valeria Parlatini[2], Tara Safa[3], Benjamin Baig[2], Hitesh Shetty[3], Jacqueline Phillips-Owen[2], Vibhore Prasad [4 *], Johnny Downs[2, 3, *]

[1] Department of Psychological Medicine, Institute of Psychiatry, Psychology and Neuroscience, Kings College London, London, UK. KING'S COLLEGE LONDON

[2] Department of Child and Adolescent Psychiatry, Institute of Psychiatry, Psychology and Neuroscience, Kings College London, London, UK KING'S COLLEGE LONDON

[3] National Institute for Health Research (NIHR) Biomedical Research Centre, South London and Maudsley NHS Foundation Trust, London, UK.

[4] School of Population Health and Environmental Sciences, King's College London, London. UK

*Contributed equally

Corresponding author: valeria.parlatini@kcl.ac.uk

Table S1. ICD-10 coding framework used for HES data extraction for physical health characteristics within inpatient episode discharge diagnoses codes.

Physical Health Characteristics	ICD10 codes
Autoimmune conditions	D81, D82.1, D83, D89.1, E31.0
CNS infections	A17, A81.1, B20-24, G00-09
CNS myo-degenerative diseases	G23.0, G10, G11, G12, G23, G31, G35-G37, G46, G60, G70-G73, G80, G91, G93.1, G93.4, G93.6, G93.7-G93.9, G94, G96, G98
Congenital malformations, deformations and chromosomal abnormalities	Q00-Q07, Q75, Q86, Q85, Q87, Q90-Q99, F84.2, F84.3
Endocrine, nutritional and metabolic diseases	E34.8, E70.2, E71, E72, E74, E75, E76, E77, E79, E83.0, E88.0, E88.1
Neoplasms	C00-C97, D32, D33, D43, D44.4, D48
Perinatal complications	P05-P08, P10, P11.0-11.2, P35-P39, P91-P96

Covariates

CRIS data was used to provide additional demographic and clinical information including sex, age at first coding of ASD diagnosis in the CAMHS records, comorbid ID (ICD-10 F7x), family history of epilepsy, ethnicity, and neighbourhood levels of socio-economic deprivation. ID was incorporated as a possible confounder due to its high correlation with both epilepsy and ADHD. History of first degree relative with epilepsy was derived from clinical note reviews of the family history section in clinical assessment documents. This has been incorporated as familial epilepsy is associated with an increased risk of offspring ADHD diagnosis [1]. Ethnicity was defined according to the guidelines of the UK Office for National Statistics. Similarly, at the time of data extraction from CRIS, neighbourhood levels of socio-economic deprivation were derived from Index of Multi-deprivation (IMD) scores provided by the UK Office of National Statistics [2], as an aggregate of income, employment, health and disability, education, skills and training, barriers to housing and services, living environment, and crime for the corresponding residential area [3].

References

1. Brikell, I., et al., *Familial Liability to Epilepsy and Attention-Deficit/Hyperactivity Disorder: A Nationwide Cohort Study.* Biological psychiatry, 2018. **83**(2): p. 173-180.
2. Leeser, R., *English Indices of Deprivation 2010: A London Perspective.* , C.L.G.M. Ministry of Housing, Editor. 2011.
3. UK Department for Communities and Local Government, *The English Indices of Deprivation 2010*. 2011.
